# Social exclusion and mobile phone dependence in college students: a moderated mediation model of self-control and social self-efficacy

**DOI:** 10.3389/fpsyg.2026.1752086

**Published:** 2026-04-13

**Authors:** Li Lv, Jialin Huang, Yingchun Cao

**Affiliations:** Department of Psychology, School of Normal, Hubei University, Wuhan, China

**Keywords:** college students, mobile phone dependence, self-control, social exclusion, social self-efficacy

## Abstract

This study integrated Compensatory Internet Use Theory with the I-PACE model to examine a moderated mediation model, aiming to understand the psychological processes linking social exclusion and mobile phone dependence among college students. A cross-sectional survey of 438 Chinese college students (after data cleaning) using validated scales revealed three key findings: First, social exclusion was positively associated with mobile phone dependence; Second, self-control partially mediated this association. Third, social self-efficacy moderated both the direct link between social exclusion and dependence and the first stage of the mediating pathway (from social exclusion to self-control). Consistent with the Expectancy Violation Theory, the positive correlation between social exclusion and mobile phone dependence was stronger among students with higher (vs. lower) social self-efficacy. These findings reveal complex mechanisms underlying mobile phone dependence, highlighting the mediating role of self-control and the counter-amplifying effect of social self-efficacy. Thus, while self-control training may offer broad benefits, tailored interventions should be designed for individuals with high social self-efficacy. Such interventions should guide their social confidence toward repairing offline relationships rather than retreating into online compensatory behaviors.

## 1 Introduction

### 1.1 Background and significance of the study

Recent national statistics indicate that over 99% of Chinese internet users access the web via mobile devices [China Internet Network Information Center (CNNIC), 2024], underscoring the centrality of smartphones in daily life. Currently, smartphones serve a multitude of purposes in daily life, including social contact, communication, information access, and entertainment. Dependence on mobile phones, the primary means of internet access, has consequently emerged as a significant public health issue. Given their ample leisure time and diverse daily needs, such as e-commerce, social interactions, and online study, college students constitute a significant proportion of users. Consequently, smartphones occupy a central role in the daily lives of college students. The mobile usage trends of college students have been empirically established by domestic studies. According to Zhao’s (2014) survey of 719 students, which combined questionnaires and interviews, almost half of them used their phones for 2–4 h per day, with 10 % spending more than 5 h and there was no discernible difference in this usage time between grade levels. According to Rong (2018) survey of 9,781 college students from 2,240 institutions across China, nearly 70% showed signs of mobile phone dependence.

Dependence on mobile phones is associated with multiple adverse outcomes. Excessive smartphone use may have negative effects on both physical and mental health, including difficulty concentrating, reduced learning efficiency, and disruption to daily routines. For instance, using phones late at night may lead to oversleeping, thereby lowering energy levels and quality of life. According to research by He et al. (2022), teenage mental health is considerably predicted negatively by mobile phone dependence. Additional research has also linked college students’ dependence on cell phones to social anxiety, sleeplessness, and rejection sensitivity (Zhang, 2021). College students’ daily lives are fragmented by frequent phone use, which continually diverts attention and makes it difficult to concentrate on studies. In practical contexts, this shows itself as a large number of students using their phones for social media, gaming, shopping, reading novels, and other activities during class. Therefore, investigating the underlying causes of mobile phone dependence among college students is crucial, as it can lead to significant academic impairment.

College students frequently encounter social exclusion, an interpersonal stressor that thwarts fundamental psychological needs for belonging and relatedness. Basic psychological demands for connection and belonging are threatened by social exclusion (Baumeister and Leary, 1995; Deci and Ryan, 2000). Self-Determination Theory states that one of the three basic psychological needs necessary for wellbeing is relatedness, or the need to connect with others (Ryan and Deci, 2017). People are compelled to look for alternate means of connection when social exclusion thwarts this urge, which may include smartphone-accessible online social networks. Through the community-building features and interactive convenience of the internet, using a phone offers companionship and makes it easier to discover like-minded online pals. People with different interests might create unique social circles because to these characteristics. Through their phones, individuals can actively seek out content aligned with their interests, thereby fostering a sense of community in online spaces.

According to pertinent studies, social exclusion is positively associated with the incidence of deviant online activities (Wang et al., 2020). Social exclusion is one of the key factors associated with mobile phone dependence, and more research is necessary to understand its underlying mechanisms. This study therefore aims to investigate the association between social exclusion and mobile phone dependence among college students and its underlying mechanisms, given the prevalence of both social exclusion and mobile phone dependence among this demographic. In order to create a moderated mediation model, this study creatively includes two intrinsic psychological variables: social self-efficacy (as positive psychological capital) and self-control (as a protective factor). This approach seeks to methodically identify the intricate processes by which social exclusion influences dependence on mobile phones. In addition to facilitating a more thorough understanding of the psychological mechanisms connecting social exclusion to mobile phone dependence, this research perspective offers specific theoretical underpinnings and useful intervention targets for upcoming behavioral and psychological interventions aimed at university students.

### 1.2 Research status

#### 1.2.1 Social exclusion and mobile phone dependence: from direct effects to complex mechanisms

Social exclusion refers to the experience of being rejected or ignored by others, threatening fundamental psychological needs for belonging and connection (Baumeister and Leary, 1995; Williams, 2006). Extensive research has established a positive correlation between social exclusion and problematic mobile phone use, typically explained by the compensatory internet use theory (Kardefelt-Winther, 2014): individuals turn to online platforms to compensate for unmet offline social needs (Hu et al., 2017; Xu et al., 2021). However, recent international research has moved beyond direct effects to explore the complex mechanisms underlying this connection. For instance, research has shown that neural sensitivity to social exclusion is associated with physiological markers such as resting blood pressure (Dembling et al., 2025), highlighting the connection between neural and physiological responses. Furthermore, research indicates that individual differences such as narcissism (Büttner et al., 2025) and self-concept (Wu et al., 2025) significantly moderate how people experience and respond to social exclusion. Studies on the bystander effect also demonstrate that even indirect exposure to exclusion influences observers’ emotional and physiological responses (Poon and Chan, 2025). These studies underscore the complexity of social exclusion’s impact and the necessity of considering individual differences and contextual factors.

#### 1.2.2 Mediating role of self-control

Mediation models have been widely employed to identify pathways from social exclusion to mobile phone dependence. Self-control has emerged as a key mediating factor, consistent with the finite resources model (Baumeister et al., 1998; Baumeister et al., 2005). Research indicates that social exclusion depletes self-control resources, thereby increasing susceptibility to problematic mobile phone use (Yao, 2023; Liang et al., 2020). However, recent research has expanded the range of examined mediating factors. For instance, loneliness (Erzen and Çikrikci, 2018), fear of missing out (FoMO; Elhai et al., 2020; Elhai et al., 2021), and social anxiety (Marino et al., 2018) have been identified as intervening variables in the exclusion-to-dependence relationship. Furthermore, chained mediation models have been proposed, linking social exclusion to mobile phone dependence through loneliness and FoMO (see, e.g., Fang et al., 2020, for a related chain mediation in the digital context). While these single and chained mediation models advance our understanding of what mediates this relationship, they fail to address the question of for whom these pathways are most significant. As recent research demonstrates, individual characteristics such as narcissism (Büttner et al., 2025), self-construal (Wu et al., 2025), and personality traits (Takeda et al., 2025) significantly moderate how people experience and respond to social exclusion. Moreover, longitudinal studies have shown that prolonged exposure to social exclusion may contribute to the development of maladaptive personality traits (Pu and Gan, 2025), suggesting a bidirectional relationship between personal characteristics and exclusion experiences. This underscores the necessity of testing moderated mediation frameworks to examine conditional indirect effects.

#### 1.2.3 Moderating role of social self-efficacy

The role of social self-efficacy—an individual’s confidence in their ability to handle social situations—has long been a subject of theoretical debate. From a social cognitive perspective, self-efficacy beliefs buffer against adversity (Bandura, 1997). Consistent with this, individuals with high social self-efficacy report better psychological adaptation (Wei et al., 2005) and lower mobile phone dependence (Xu et al., 2021). However, emerging research challenges this unified protective view. Studies on rejection sensitivity suggest that individuals highly sensitive to social feedback may be more vulnerable to interpersonal stressors (Downey and Feldman, 1996; Gao et al., 2017). The “too much of a good thing” effect (Pierce and Aguinis, 2013) suggests positive traits may exhibit nonlinear or context-dependent effects, becoming maladaptive under specific conditions. In the digital realm, Valkenburg et al. (2021) found adolescents with stronger social skills engaged in more proactive social media use and were more sensitive to feedback, potentially increasing vulnerability to online rejection. Recent neuroimaging evidence further supports the moderating role of individual differences in social processing on responses to rejection. For instance, studies indicate that neural sensitivity to rejection varies across individuals depending on personality traits (Büttner et al., 2025; Zhang X. et al., 2025), and physiological responses to social stress are modulated by individual characteristics (Dembling et al., 2025). Thus, whether social self-efficacy buffers or amplifies the impact of social exclusion remains an open question.

#### 1.2.4 Moderators of the exclusion–dependence link

Beyond mediating factors, the study also identified moderators influencing the strength of the rejection-dependence relationship. Rejection sensitivity (Downey and Feldman, 1996; Gao et al., 2017) and individual difference factors such as narcissism (Büttner et al., 2025), growth mindset (Yang et al., 2025), and relationship orientation (Lu et al., 2025) have been shown to moderate the effects of social rejection. These findings underscore the importance of considering person-level characteristics in understanding responses to exclusion. For instance, individuals with high rejection sensitivity may exhibit stronger negative reactions to rejection, leading to greater compensatory mobile phone use. However, most studies examine moderators in isolation, failing to integrate them into a unified moderated mediation framework to investigate whether the mediating paths themselves are moderated by individual differences. This gap is critical because theoretical models like the I-PACE model (Brand et al., 2019) posit that personal factors (e.g., social self-efficacy) interact with situational stressors (e.g., social exclusion) to influence cognitive and behavioral outcomes. Recent research on workplace exclusion further emphasizes the role of individual differences in shaping exclusionary responses, but its application in the field of digital dependence remains to be explored.

Recent research has expanded our understanding of the mechanisms linking social exclusion and problematic mobile phone use. For instance, some studies have identified loneliness (Erzen and Çikrikci, 2018), fear of missing out (Elhai et al., 2020), and social anxiety (Marino et al., 2018) as mediating factors, while others have emphasized moderating factors such as rejection sensitivity (Gao et al., 2017) and narcissism (Büttner et al., 2025). However, these studies typically examine mediation or moderation in isolation, without considering whether mediating pathways depend on individual characteristics. Although recent research has begun exploring moderated mediation models in related domains (e.g., Fang et al., 2020 on screen-gazing behavior; Xu et al., 2021 on social anxiety), no study has specifically tested whether the indirect effect of social exclusion on phone dependence via self-control is moderated by social self-efficacy. Furthermore, competing theoretical predictions regarding the role of social self-efficacy remain unexamined. Therefore, a unified moderated mediation model simultaneously testing mediation and moderation is needed to enhance our understanding of the complex processes underlying mobile phone dependence.

#### 1.2.5 Summary of gaps and the present study

In summary, although prior research has established the link between social exclusion and mobile phone dependence through mediating factors such as self-control and moderating factors such as rejection sensitivity, key gaps remain. First, few studies have integrated mediation and moderation into a unified moderated mediation framework to examine whether the indirect effect of exclusion on dependence via self-control is moderated by levels of social self-efficacy. Second, the role of social self-efficacy under interpersonal stress remains a theoretical puzzle. Social Cognitive Theory (Bandura, 1997) posits that self-efficacy beliefs buffer individuals from adversity, suggesting that high social self-efficacy should attenuate the link between social rejection and adverse consequences (the buffering hypothesis). Conversely, the Expectancy Violation Theory (Burgoon, 1993) suggests that individuals with strong positive expectations of social acceptance may experience greater psychological distress when these expectations are violated, potentially amplifying the negative effects of exclusion (amplification hypothesis). These two theoretical perspectives yield directly opposing predictions, yet no empirical research has systematically tested them in the context of mobile phone dependence. This unresolved theoretical tension limits our understanding of when and for whom social self-efficacy fosters resilience or vulnerability following social exclusion. Third, recent international research has revealed the complexity of social exclusion effects (e.g., studies on neural and physiological responses to exclusion, such as Zhang X. et al., 2025, Zhang J. et al., 2025; Dembling et al., 2025; and studies on individual difference moderators, such as Büttner et al., 2025; Wu et al., 2025). However, these insights have yet to be integrated into mobile phone dependence models. This study addresses these gaps by proposing and testing a moderated mediation model that simultaneously examines (a) the mediating role of self-control and (b) whether this mediation is moderated by social self-efficacy. By pitting the buffering hypothesis against the amplification hypothesis, we aim to empirically adjudicate two theoretically plausible yet opposing explanations for how these personal resources operate under interpersonal stress.

### 1.3 Theoretical framework of this study

To comprehensively understand the mechanisms linking social exclusion to mobile phone dependence, we integrate two complementary theoretical perspectives: Compensatory Internet Use Theory (CIUT; Kardefelt-Winther, 2014) and the I-PACE model (Brand et al., 2019). CIUT explains the motivation for compensatory behavior by proposing that when psychological needs (e.g., belongingness) are thwarted in offline contexts, individuals seek alternative fulfillment online (Kardefelt-Winther, 2014). The I-PACE model, in turn, provides a process-oriented framework specifying how personal characteristics and situational factors interact to produce addictive behaviors (Brand et al., 2019).

Within this integrated framework, social exclusion is conceptualized as an environmental stressor, with social self-efficacy serving as the core personal factor (P) that shapes individuals’ perceptions and responses to exclusion. Although our cross-sectional design did not directly measure affective states, we theoretically hypothesize that social exclusion elicits negative emotional reactions (A)—such as feelings of rejection, loneliness, or hurt—which may precede subsequent cognitive and behavioral processes. This hypothesis remains theoretical and warrants direct examination in future research.

Regarding self-control, we draw on the finite resource model (Baumeister et al., 1998) to construct theoretical reasoning. However, it must be clarified that we measure trait self-control (Tangney et al., 2004; Tan and Guo, 2008), reflecting relatively stable individual differences in self-regulatory capacity. Trait self-control should be regarded as a dispositional resource, aligning more closely with the personal factor (P) in the I-PACE model rather than with the momentary executive function (E). Within the I-PACE framework, executive function (E) refers to dynamic processes involving inhibitory control and decision-making that may be depleted under stress. Since we measured trait self-control, our findings do not indicate immediate resource depletion. Instead, they suggest that social exclusion may be associated with lower trait self-control through long-term mechanisms, or that individuals with low trait self-control may be more susceptible to perceiving exclusion. Future research should employ experimental or longitudinal designs to examine the dynamic interaction between exclusion and self-regulatory resources.

Furthermore, a critical distinction must be made between executive functions and behavioral outcomes: in the I-PACE model, the Execution dimension (E) refers to processes, not outcomes (Brand et al., 2019). Consistent with this specification, we treat self-control as an executive function process (E), whereas mobile phone dependence is conceptualized as the behavioral outcome, resulting from the interplay among person factors, affective responses, cognitive processing, and executive functions. This distinction follows recent extensions of the I-PACE model that separate executive functions from the behaviors they regulate (Brand et al., 2019, p. 5).

In summary, our integrated framework positions social exclusion as an environmental stressor, social self-efficacy as a personal factor (P), self-control as a dispositional resource (which we classify under the P dimension rather than the dynamic E dimension), and mobile phone dependence as a behavioral outcome. We acknowledge that the I-PACE model also encompasses affective (A) and cognitive (C) processes, which we did not measure; thus, our testing represents a partial and simplified operationalization of the full model. We employed mediation and moderation analyses to examine predictive relationships and conditional mechanisms among measured variables, while recognizing that unmeasured affective and cognitive processes may also play significant roles. By conceptualizing self-control as a relatively stable self-regulatory tendency, we avoid confounding it with momentary executive functions. This framework helps explain why socially excluded individuals may turn to phones for compensation and reveals where this compensatory process is most pronounced—specifically, whether the self-control pathway is moderated by levels of social self-efficacy.

Based on the integrated theoretical framework and the competing predictions derived from EVT and Social Cognitive Theory, we test the following hypotheses, which include both relatively established relationships (H1, H2) and competing predictions regarding the moderating role of social self-efficacy (H3, H4):

*H1*: Social exclusion is positively associated with mobile phone dependence among college students.

*H2*: Self-control mediates the relationship between social exclusion and mobile phone dependence.

Drawing from Expectancy Violation Theory, we propose the amplification hypothesis for the moderating role of social self-efficacy. Specifically, we hypothesize (H3) that social self-efficacy moderates the relationship between social exclusion and self-control, such that the negative effect of exclusion on self-control is stronger among students with high social self-efficacy. This prediction stands in contrast to the buffering hypothesis derived from Social Cognitive Theory, which suggests that high social self-efficacy should attenuate the negative effect.

*H3*: Social self-efficacy moderates the relationship between social exclusion and self-control. Based on the competing theoretical frameworks outlined above, we propose two competing predictions: (a) the buffering hypothesis (derived from Social Cognitive Theory) predicts that social self-efficacy attenuates the negative effect of exclusion on self-control; (b) the amplification hypothesis (derived from Expectancy Violation Theory) predicts that social self-efficacy exacerbates this negative effect. Given the theoretical tension between these perspectives, we do not make a directional prediction but rather let the data adjudicate between them.

Similarly, drawing from Expectancy Violation Theory, we propose the amplification hypothesis for the direct pathway. We hypothesize (H4) that social self-efficacy moderates the direct relationship between social exclusion and mobile phone dependence, such that the positive association between social exclusion and mobile phone dependence is stronger for students with high social self-efficacy. Again, this competes with the buffering hypothesis derived from Social Cognitive Theory.

*H4*: Social self-efficacy moderates the direct relationship between social exclusion and mobile phone dependence. Again, we propose two competing predictions: (a) the buffering hypothesis predicts that social self-efficacy weakens the positive association between exclusion and dependence; (b) the amplification hypothesis predicts that social self-efficacy strengthens this association.

## 2 Research methodology

### 2.1 Participants

The study was performed in compliance with relevant laws and institutional guidelines and has been approved by the Department of Psychology, School of Education, Hubei University (Ethical Approval Number 20231013). The privacy rights of all participants were protected, and an informed consent agreement was reached with all participants before they filled out the questionnaire. A convenience sample of 500 Chinese university students was recruited through the Wenjuanxing platform. Participants were from multiple universities across different regions, majoring in varied disciplines including sciences, humanities, and social sciences. A total of five hundred questionnaires were distributed and collected. To ensure data quality, four attention check items are embedded in the scales: Item 14 of the Social Exclusion Scale requires selecting “Sometimes” (coded as 3); Item 8 of the Mobile Phone Addiction Index requires selecting ‘Always’ (5); Item 19 of the Self-Control Scale requires selecting “Strongly Disagree” (1); Item 13 of the Social Self-Efficacy Scale requires selecting “Strongly Agree” (5) Participants who answered all four check items incorrectly were deemed invalid samples and excluded. This criterion led to the removal of 62 questionnaires, leaving a final valid sample of 438 participants (effective response rate = 87.6%). There were 244 female students (55. 7%) and 194 male students (44. 3%) in the sample. Forty-two students (9. 6%) were freshmen. A total of 155 students (35. 4%) are sophomores. 162 students (37. 0%) are juniors. 79 students (18. 0%) are seniors. We acknowledge that applying strict criteria (all incorrect answers) may exclude some participants who occasionally erred but were genuinely committed. As a sensitivity analysis, we also re-ran the primary analysis using more lenient criteria (at least three incorrect answers); the pattern of results remained unchanged, supporting the reliability of this study’s findings.

Demographic variables are coded as follows: gender (1 = male, 2 = female), place of origin (1 = rural, 2 = urban), major (1 = humanities/social sciences, 2 = science/engineering, 3 = other), and academic year (1 = freshman, 2 = sophomore, 3 = junior, 4 = senior). All primary analyses include these variables as covariates to control for potential confounding effects.

### 2.2 Tools

#### 2.2.1 Social Exclusion Scale

Wu et al.’s (2013) Social Exclusion Questionnaire for College Students was used. This 19-item measure assesses two aspects: indirect exclusion (e.g., “When I feel down, I receive no consolation or comfort from others”) and direct exclusion (e.g., “When others tease or playfully fight, they intentionally or unintentionally avoid me”). It evaluates people’s experiences of social exclusion in day-to-day life using a 5-point Likert scale, where 1–5 is “Never—Often. “Greater social exclusion is indicated by higher scores on the scale, which has 10 items for direct exclusion and 9 items for indirect exclusion. The scale demonstrated excellent internal consistency (Cronbach’s *α* = 0.96) and satisfactory construct validity in prior validation studies.

#### 2.2.2 Mobile Phone Addiction Index

The Chinese version of the Mobile Phone Addiction Index (MPAI), translated and revised by Huang et al. (2013), was used. This scale measures what this paper refers to as the construct of mobile phone dependence. This 17-item scale (e.g., “You have been told that you spend too much time on your phone”) was developed by Leung (2008) at The Chinese University of Hong Kong to assess college students’ addiction to mobile phones. Loss of control, disengagement, escape, and inefficiency are its four components. A 5-point Likert scale is used for scoring, with 1–5 representing “Never–Very often.” The total score ranges from 17 to 85 points. Dependence on mobile phones is indicated by a score greater than 51, where higher scores imply greater dependence. The validity and reliability of the scale are good. In this investigation, the Cronbach’s *α* coefficient was 0. 94.

#### 2.2.3 Self-Control Scale

Tan and Guo (2008) updated College Student Self-Control Scale (SCS) was employed. The Self-Control Scale was originally developed by Tangney et al. (2004) and later adapted for Chinese college students by Tan and Guo (2008). Five dimensions—impulse control, healthy behaviors, resisting temptation, job focus, and entertainment restraint—are covered by the 19 items in the Chinese version. It has 15 reverse-scoring items and 4 forward-scoring items on a 5-point Likert scale, with 1 denoting “strongly disagree–strongly agree. “The scores of forward-scored things are increased by the scores of reversed items. Stronger self-control skills are indicated by higher scores. In this investigation, the Cronbach’s α coefficient was 0. 94.

#### 2.2.4 Social Self-Efficacy Scale

We used the Social Self-Efficacy Scale, which was created by Smith and Betz (2000) and updated by Gu et al. (2014). The revised Chinese version consists of 18 items rated on a 5-point Likert scale (1 = no confidence at all, 5 = complete confidence). As scores rise on this unidimensional scale, social self-efficacy increases. In this study, Cronbach’s α was 0. 95.

Scoring and Standardization. For descriptive purposes, we report both total scores (to facilitate comparison with established clinical cut-offs) and mean item scores (to enable comparability across scales). All primary analyses (correlations, regressions, mediation, and moderated mediation) were conducted using mean item scores, which place all variables on a consistent 1–5 metric and facilitate interpretation of regression coefficients. Total scores were obtained by summing item responses, while mean scores were calculated by dividing the total score by the number of items in each scale.

### 2.3 Data processing

Data analysis was conducted using SPSS 27.0 and the PROCESS macro version 4.1. We examined the mediation model (Model 4) and the moderated mediation model (Model 8). Gender, grade level, and place of origin were included as covariates in the models because existing literature indicates these demographic factors may be systematically associated with adolescents’ quality of social relationships, psychological resources, and media usage habits (e.g., Marino et al., 2018; Wei et al., 2005). Including them in the model helped isolate relatively “pure” associations between social exclusion, self-control, and social self-efficacy, reducing the influence of potential confounders. To mitigate multicollinearity, all predictor variables were mean-centered prior to constructing interaction terms. The significance of indirect and interaction effects was tested using bias-corrected bootstrap confidence intervals based on 5,000 resamples. An effect was considered significant if its 95% confidence interval did not include zero.

Following recommendations for transparent reporting, we present unstandardized coefficients (b), standard errors (SE), *t*-values, *p*-values, and 95% confidence intervals for all regression models (see Tables 1, 2). For indirect effects, we report bootstrapped standard errors and bias-corrected confidence intervals (see Tables 1, 3). Standardized coefficients (*β*) are also provided in Table 2 for comparability with prior research.

**Table 1:** Mediation analysis results: self-control as mediator.

Path	Unstd. *b*	SE	*t*-value	*p*-value	Effect size	95% CI (5,000 samples)
Total effect	0.58	0.043	9.67^***^	0.000		[0.49, 0.67]
Direct effect (social exclusion → mobile phone dependence)	0.29	0.044	6.47^***^	0.000	50%	[0.21, 0.38]
Mediation effect (social exclusion → self-control → mobile phone dependence)	0.29	0.034	8.76^***^	0.000	50%	[0.22, 0.36]

Bootstrap resamples = 5,000; controlled for gender, grade, and hometown; all continuous variables are based on mean scores (total score divided by number of items).

***p < 0.001.

**Table 2:** Regression coefficients for the moderated mediation model.

Outcome variable	Predictor variable	Unstd. *b*	SE	Std. *β*	*t*	*p*	95% CI
Self-control	Gender (control)	−0.52	0.72	−0.03	−0.72	0.471	[−1.94, 0.90]
	Grade (control)	−1.89	0.75	−0.11	−2.52^*^	0.012	[−3.37, −0.41]
	Hometown (control)	−1.35	0.71	−0.08	−1.89	0.059	[−2.75, 0.05]
	Social exclusion	−0.36	0.05	−0.36	−7.84^***^	<0.001	[−0.45, −0.27]
	Social self-efficacy	0.38	0.04	0.38	8.90^***^	<0.001	[0.30, 0.46]
	Interaction	−0.14	0.04	−0.14	−3.19^**^	0.001	[−0.22, −0.06]
	Gender (control)	0.89	0.75	0.05	1.18	0.238	[−0.59, 2.37]
Mobile phone dependence	Grade (control)	−1.21	0.73	−0.07	−1.65	0.100	[−2.65, 0.23]
	Hometown (control)	1.03	0.75	0.06	1.37	0.172	[−0.45, 2.51]
	Social exclusion	0.29	0.04	0.29	6.47^***^	<0.001	[0.21, 0.37]
	Self-control	−0.49	0.04	−0.49	−11.28^***^	<0.001	[−0.57, −0.41]
	Social self-efficacy	−0.10	0.04	−0.10	−2.42^*^	0.016	[−0.18, −0.02]
	Interaction	0.09	0.04	0.09	2.29^*^	0.022	[0.01, 0.17]

^*^p < 0.05, ^**^p < 0.01, ^***^p < 0.001. All continuous variables are based on mean scores (total score divided by number of items).

**Table 3:** Conditional indirect effects at different levels of social self-efficacy.

Level of social self-efficacy	Indirect effect	SE	95% CI
Low (*M* − 1SD)	0.12	0.03	[0.07, 0.18]
Medium (*M*)	0.18	0.02	[0.14, 0.22]
High (*M* + 1SD)	0.23	0.03	[0.17, 0.29]
Index of moderated mediation	0.07	0.02	[0.02, 0.12]

All variables are based on mean scores (total score divided by number of items).

To minimize the potential impact of common method bias, we implemented multiple procedural controls: (a) assuring participants of the anonymity and confidentiality of their responses; (b) including reverse-scored items in the questionnaire to reduce response patterns; (c) randomizing the order of scale presentation across participants. Additionally, we conducted a Harman single-factor test and confirmatory factor analysis (see Section 3.1), which indicated that common method bias was unlikely to be a significant concern.

Given the cross-sectional design of this study, causal conclusions cannot be drawn. This analysis aims to examine the hypothesized relationships and conditional effects among variables within the theoretical model. These relationships should be further tested in future longitudinal or experimental studies to determine temporal sequence and causality.

### 2.4 Multicollinearity diagnostics

To examine potential multicollinearity, we calculated variance inflation factors (VIF) for all predictors in the regression models. As shown in Table 4, all VIF values were below the conventional threshold of 10, ranging from 1.05 to 3.11. These results indicate that multicollinearity is not a serious concern in this study.

**Table 4:** Variance inflation factors for multicollinearity diagnostics.

Model	Variable	VIF	Model	Variable	VIF
Model 1 (predicting self-control)	Social exclusion	1.41	Model 2 (predicting mobile phone dependence)	Social exclusion	1.62
	Social self-efficacy	1.30		Self-control	1.65
	Interaction	1.05		Social self-efficacy	1.53
	Gender (male)	1.06		Interaction	1.08
	Grade (sophomore)	3.08		Gender (male)	1.07
	Grade (junior)	3.11		Grade (sophomore)	3.09
	Grade (Senior)	2.40		Grade (junior)	3.11
	Hometown (Urban)	1.05		Grade (senior)	2.40
	–	–		Hometown (urban)	1.05

All VIF values are well below the cutoff of 10, indicating no serious multicollinearity. All continuous variables are based on mean scores.

## 3 Research findings

### 3.1 Control and examination of common method bias

To assess common method bias, we conducted a Harman single-factor test following the procedure recommended by Zhou and Long (2004). Results indicated that the first unrotated factor explained 34.39% of total variance, below the recommended threshold of 40%, suggesting that common method bias is unlikely to be a significant issue in this study (see Table 5).

**Table 5:** Common method bias test results.

Fit index	Value
Kaiser–Meyer–Olkin measure of sampling adequacy	0.960
Bartlett’s test of sphericity	*χ*^2^ (2,628) = 23,316.00, *p* < 0.001
Variance explained by the first factor	34.39%
*X*^2^	13,187.868
df	2,555
*X*^2^/df	5.16
CFI	0.520
TLI	0.507
RMSEA	0.097
95%CI for RMSEA	[0.096, 0.099]
SRMR	0.120

*N* = 438. Estimation method = Maximum Likelihood (ML). The one-factor model showed poor fit (CFI < 0.90, RMSEA > 0.08, SRMR > 0.08), indicating that common method bias is unlikely to be a serious concern.

To provide stronger evidence, we further performed a confirmatory factor analysis (CFA) with a single-factor model where all items were loaded onto one common factor. As shown in Table 5, the one-factor model exhibited poor fit to the data (*χ*^2^/df = 5.16, CFI = 0.520, TLI = 0.507, RMSEA = 0.097, SRMR = 0.120), with all fit indices falling below acceptable thresholds. The poor fit of the single-factor model provides additional evidence that common method bias is not a serious concern in this study.

### 3.2 Demographic differences in variables

#### 3.2.1 Sample characteristics

The demographic characteristics of the final sample (*N* = 438) are presented in Table 6. The sample consisted of 194 male (44.3%) and 244 female (55.7%) participants. In terms of hometown, 211 participants (48.2%) were from rural areas and 227 (51.8%) from urban areas. Regarding academic major, 179 students (40.9%) were from humanities/social sciences, 240 (54.8%) from science/engineering, and 19 (4.3%) from other disciplines. By grade level, there were 42 freshmen (9.6%), 155 sophomores (35.4%), 162 juniors (37.0%), and 79 seniors (18.0%).

**Table 6:** Descriptive statistics for all study variables (unified total score and item mean).

Variable	Total score range	*N*	Total score (*M* ± SD)	Item mean (*M* ± SD)
Social exclusion	19–95	438	38.89 ± 15.43	2.04 ± 0.81
Mobile phone dependence	17–85	438	51.00 ± 14.79	3.00 ± 0.87
Self-control	19–95	438	60.61 ± 15.95	3.19 ± 0.85
Social self-efficacy	18–90	438	60.66 ± 15.48	3.37 ± 0.86

#### 3.2.2 Social exclusion

Differences in social exclusion across demographic variables (gender, hometown type, major category, grade level) are examined in Table 7.

**Table 7:** Differences in social exclusion by demographic variables.

Variable	Category	*N*	Total score (*M* ± SD)	*t*/*F*	*p*
Social exclusion	Male	194	40.90 ± 15.98	2.58^**^	0.010
	Female	244	37.11 ± 14.73		
	Rural	211	40.95 ± 16.03	2.86^**^	0.004
	Urban	227	36.78 ± 14.53		
	Humanities/social sciences	179	38.94 ± 15.13	0.22	0.809
	Science/engineering	240	38.85 ± 15.74		
	Other disciplines	19	36.53 ± 13.92		
	Freshman	42	38.90 ± 14.14	3.81^**^	0.010
	Sophomore	155	41.08 ± 15.80		
	Junior	162	38.92 ± 16.16		
	Senior	79	33.96 ± 12.53		

^*^p < 0.05, ^**^p < 0.01. Coding: Gender (1 = Male, 2 = Female); Hometown (1 = Rural, 2 = Urban); Major (1 = Humanities, 2 = Science, 3 = Other); Grade (1 = Freshman, 2 = Sophomore, 3 = Junior, 4 = Senior). Post-hoc tests (Tukey HSD) indicated a significant difference between sophomore and senior (*p* = 0.0045). All analyses are based on mean scores (total score divided by number of items).

Males (*M* = 40.90, SD = 15.98) scored significantly higher than females (*M* = 37.11, SD = 14.73), *t* = 2.58, *p* = 0.010. Rural students (*M* = 40.95, SD = 16.03) reported higher social exclusion than urban students (*M* = 36.78, SD = 14.53), *t* = 2.86, *p* = 0.004. No significant differences emerged across major categories, *F* (2, 435) = 0.22, *p* = 0.809. Grade level showed significant variation, *F* (3, 434) = 3.81, *p* = 0.010; post-hoc tests (Tukey HSD) revealed that sophomores (*M* = 41.08, SD = 15.80) and juniors (*M* = 38.92, SD = 16.16) scored significantly higher than seniors (*M* = 33.96, SD = 12.53), *p* = 0.0045.

#### 3.2.3 Testing differences in variables by hometown type

Mobile phone dependence, self-control, and social self-efficacy across demographic characteristics were examined using independent samples t-tests and one-way ANOVA. Only the location of origin for each of the three variables showed significant differences, as Table 8 illustrates.

**Table 8:** Differences in key variables by hometown type.

Variable	Hometown	*N*	Total score (*M* ± SD)	Item mean (*M* ± SD)	*t*-value	*p*-value
Mobile phone dependence	Rural	211	52.56 ± 14.04	3.09 ± 0.82	2.07^*^	0.039
	Urban	227	49.65 ± 15.28	2.92 ± 0.89		
Self-control	Rural	211	58.60 ± 15.64	3.08 ± 0.82	−2.53^*^	0.012
	Urban	227	62.46 ± 16.26	3.29 ± 0.86		
Social self-efficacy	Rural	211	59.17 ± 14.66	3.29 ± 0.81	−2.01^*^	0.045
	Urban	227	62.11 ± 15.95	3.45 ± 0.89		

^*^p < 0.05. All variables are based on total scores.

Rural students reported higher mobile phone dependence (total score: *M* = 52.56, SD = 14.04; item mean: *M* = 3.09, SD = 0.82) compared to urban students (total score: *M* = 49.65, SD = 15.28; item mean: *M* = 2.92, SD = 0.89), *t* = 2.07, *p* = 0.039. Rural students also exhibited lower self-control (total score: *M* = 58.60, SD = 15.64; item mean: *M* = 3.08, SD = 0.82) than urban students (total score: *M* = 62.46, SD = 16.26; item mean: *M* = 3.29, SD = 0.86), *t* = −2.53, *p* = 0.012. Additionally, rural students had lower social self-efficacy (total score: *M* = 59.17, SD = 14.66; item mean: *M* = 3.29, SD = 0.81) compared to urban students (total score: *M* = 62.11, SD = 15.95; item mean: *M* = 3.45, SD = 0.89), *t* = −2.01, *p* = 0.045.

### 3.3 Descriptive statistics and correlation analysis of variables

Table 9 presents the means, standard deviations, and Pearson correlations for all study variables, all based on mean item scores (range 1–5). Social exclusion was positively correlated with mobile phone dependence (*r* = 0.52, *p* < 0.01) and negatively correlated with self-control (*r* = −0.48, *p* < 0.01) and social self-efficacy (*r* = −0.44, *p* < 0.01). Mobile phone dependence was negatively correlated with both self-control (*r* = −0.67, *p* < 0.01) and social self-efficacy (*r* = −0.48, *p* < 0.01). Self-control and social self-efficacy were positively correlated with each other (*r* = 0.54, *p* < 0.01). These correlations provide preliminary support for the hypothesized relationships.

**Table 9:** Means, standard deviations, and correlations among study variables.

Variable	*M*	SD	1	2	3	4
1. Social exclusion	2.04	0.81	–			
2. Mobile phone dependence	3.00	0.87	0.52^**^	–		
3. Self-control	3.19	0.85	−0.48^**^	−0.67^**^	–	
4. Social self-efficacy	3.37	0.86	−0.44^**^	−0.48^**^	0.54^**^	–

All variables are based on mean scores (total score divided by number of items). **p < 0.01.

### 3.4 Testing the mediating model of self-control

Gender, grade level, and hometown type were included as control variables in the analytic model since social exclusion ratings varied by these factors, as did self-control and mobile phone dependence scores. The 95% confidence interval for the mediating impact was evaluated and computed using social exclusion as the independent variable, mobile phone dependence as the dependent variable, and self-control as the mediating variable. The findings are displayed in Table 1.

Using cross-sectional data, we examined associations consistent with our theoretical model. We first examined the total, direct, and indirect effects. Bootstrap analysis with 5,000 resamples revealed a significant total effect of social exclusion on mobile phone dependence (*b* = 0.58, SE = 0.043, *t* = 9.67, *p* < 0.001, 95% CI [0.49, 0.67]). The direct association remained significant (*b* = 0.29, SE = 0.044, *t* = 6.47, *p* < 0.001, 95% CI [0.21, 0.38]), supporting H1. Critically, the indirect effect through self-control was also significant (ab = 0.29, Boot SE = 0.034, 95% Boot CI [0.22, 0.36]), confirming that self-control serves as a partial mediator and supporting H2.

**Figure 1:**
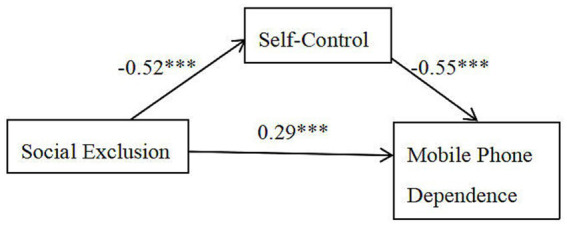
Mediating role of self-control. ***p < 0.001.

Mediation analysis revealed a significant indirect effect of social exclusion on mobile phone dependence through self-control (ab = 0.29, Boot SE = 0.034, 95% Boot CI [0.22, 0.36]), accounting for 50% of the total effect. The confidence interval did not include zero, indicating that self-control partially mediated this association. Figure 1 shows the particular mediating path.

### 3.5 Testing the moderating model of social self-efficacy

**Figure 2:**
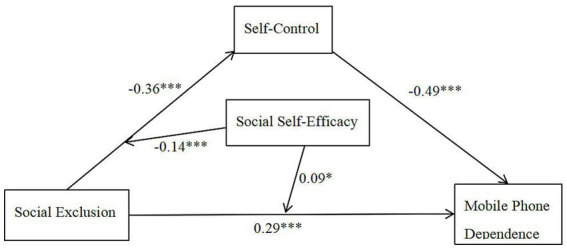
Path diagram of mediated effects with moderation. *p < 0.05, ***p < 0.001.

We tested the moderated mediation model using PROCESS Model 8 with 5,000 bootstrap samples, controlling for gender, grade, and hometown. Table 10 presents the model fit indices, and Table 2 displays the regression coefficients. The conceptual path diagram is presented in Figure 2.

**Table 10:** Model fit indices for moderated mediation analysis.

Outcome variable	*R*	*R*^2^	*F*-value	*p*-value
Self-control	0.62	0.39	45.61^***^	<0.001
Mobile phone dependence	0.72	0.52	66.69^***^	<0.001

^*^p < 0.05, ^**^p < 0.01, ^***^p < 0.001. All variables are based on mean scores (total score divided by number of items).

As shown in Table 2, social exclusion negatively predicted self-control (*b* = −0.36, SE = 0.05, *t* = −7.84, *p* < 0.001, 95% CI [−0.45, −0.27]), while social self-efficacy positively predicted self-control (*b* = 0.38, SE = 0.04, *t* = 8.90, *p* < 0.001, 95% CI [0.30, 0.46]). Importantly, the interaction term between social exclusion and social self-efficacy significantly predicted self-control (*b* = −0.14, SE = 0.04, *t* = −3.19, *p* = 0.001, 95% CI [−0.22, −0.06]). This interaction term contributed an additional 1.5% of explained variance (Δ*R*^2^ = 0.015). Although statistically significant, this effect size is relatively small, consistent with meta-analytic findings that interaction effects in social sciences are typically modest (Aguinis et al., 2021). Therefore, while the amplifying effect of social self-efficacy is reliable, its practical impact should be interpreted cautiously and warrants replication in larger samples. Thus, the data support the amplification hypothesis rather than the buffering hypothesis. Consequently, H3 (regarding the existence of a moderating effect) is supported, and its direction aligns with predictions from the Contrary Expectations Theory.

In the model predicting mobile phone dependence, self-control was a significant negative predictor (*b* = −0.49, SE = 0.04, *t* = −11.28, *p* < 0.001, 95% CI [−0.57, −0.41]), and social self-efficacy was a significant negative predictor (*b* = −0.10, SE = 0.04, *t* = −2.42, *p* = 0.016, 95% CI [−0.18, −0.02]). The direct effect of social exclusion on mobile phone dependence remained significant (*b* = 0.29, SE = 0.04, *t* = 6.47, *p* < 0.001, 95% CI [0.21, 0.37]). Notably, the interaction term between social exclusion and social self-efficacy significantly predicted mobile phone dependence (*b* = 0.09, SE = 0.04, *t* = 2.29, *p* = 0.022, 95% CI [0.01, 0.17]). The positive coefficient indicates that the positive correlation between exclusion and dependence is stronger among individuals with high social self-efficacy, further supporting the amplification hypothesis. Thus, H4 is supported, and the direction of the moderation effect aligns with the predictions of the Expectancy Violation Theory.

**Figure 3:**
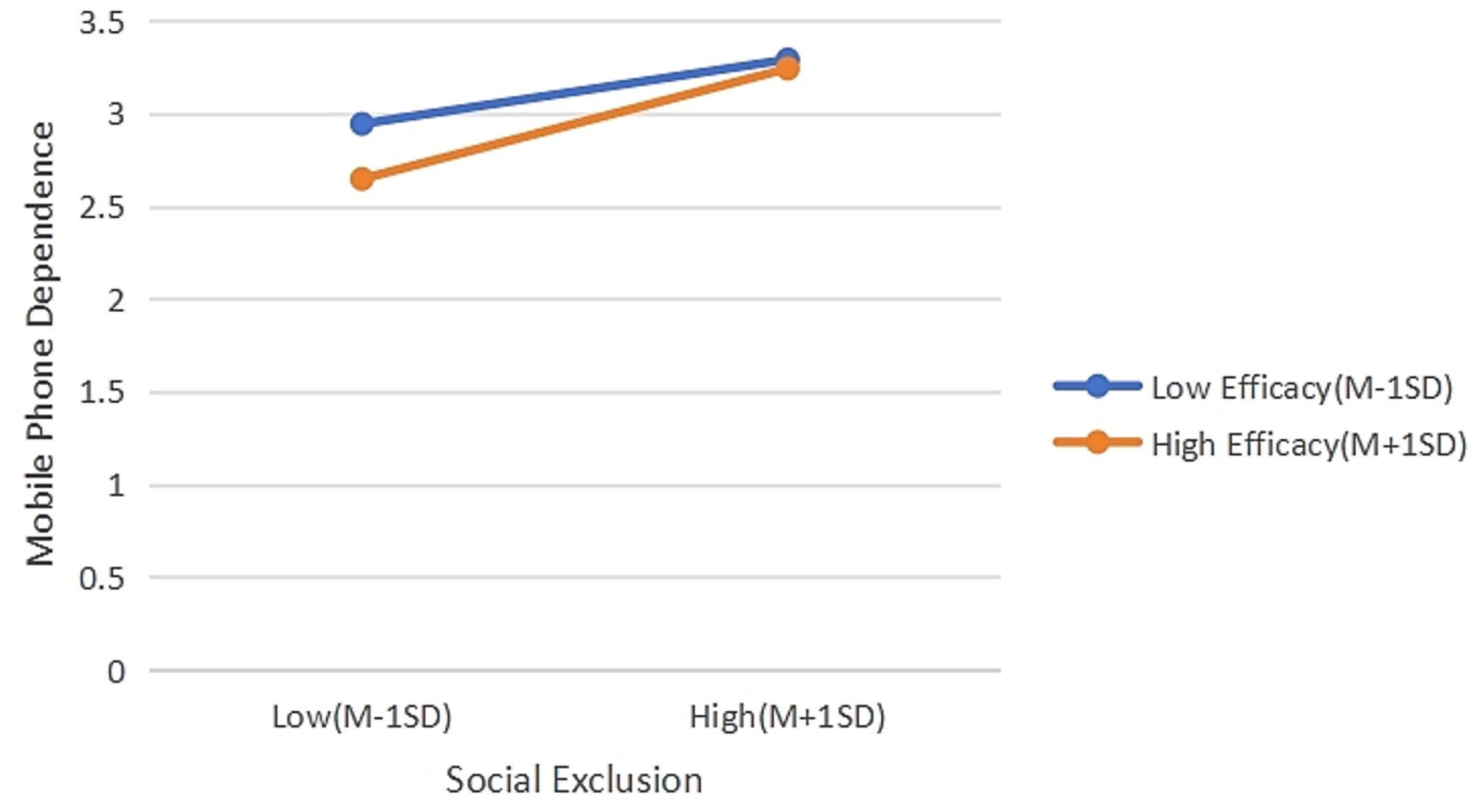
Moderating effect of social self-efficacy between social exclusion and mobile phone dependence.

Simple slope analyses were conducted to probe the significant interactions. As shown in Table 11, the positive effect of social exclusion on mobile phone dependence was stronger for individuals with high social self-efficacy (+1 SD, *b* = 0.37, SE = 0.06, *p* < 0.001) than for those with low social self-efficacy (−1 SD, *b* = 0.21, SE = 0.05, *p* < 0.001). Similarly, Table 12 shows that the negative effect of social exclusion on self-control was stronger for high SSE individuals (*b* = −0.48, SE = 0.06, *p* < 0.001) than for low SSE individuals (*b* = −0.25, SE = 0.05, *p* < 0.001). As illustrated in Figure 3, the positive association between social exclusion and mobile phone dependence was stronger for individuals with high social self-efficacy.

**Table 11:** Moderating effect of social self-efficacy on social exclusion-mobile phone dependence.

Social self-efficacy	Effect	SE	LLCI	ULCI
−0.86	0.21	0.05	0.12	0.31
0	0.29	0.04	0.20	0.38
0.86	0.37	0.06	0.24	0.49

All variables are based on mean scores (total score divided by number of items).

**Table 12:** Moderating effect of social self-efficacy on social exclusion-self-control.

Social self-efficacy	Effect	SE	LLCI	ULCI
−0.86	−0.25	0.05	−0.35	−0.14
0	−0.36	0.05	−0.45	−0.27
0.86	−0.48	0.06	−0.60	−0.35

All variables are based on mean scores (total score divided by number of items).

We further calculated the index of moderated mediation to test whether the indirect effect of social exclusion on mobile phone dependence through self-control varies across levels of social self-efficacy. As shown in Table 3, the conditional indirect effects increased from low (*b* = 0.12, Boot SE = 0.03, 95% Boot CI [0.07, 0.18]) to medium (*b* = 0.18, Boot SE = 0.02, 95% Boot CI [0.14, 0.22]) to high (*b* = 0.23, Boot SE = 0.03, 95% Boot CI [0.17, 0.29]) levels of social self-efficacy. The index of moderated mediation was significant (*b* = 0.07, Boot SE = 0.02, 95% Boot CI [0.02, 0.12]), confirming that the mediating effect of self-control is conditional on social self-efficacy.

### 3.6 Multicollinearity diagnostics

To assess potential multicollinearity among predictors, we calculated variance inflation factors (VIF) for all variables in the regression models. As detailed in Table 4, all VIF values ranged from 1.05 to 3.11, well below the conservative threshold of 5 and the conventional cutoff of 10. These results indicate that multicollinearity is not a concern in this study (Table 13).

**Table 13:** Demographic characteristics of the sample.

Variable	Category	*N*	Percentage (%)
Gender	Male	194	44.3
	Female	244	55.7
Hometown	Rural	211	48.2
	Urban	227	51.8
Major	Humanities/social sciences	179	40.9
	Science/engineering	240	54.8
	Other disciplines	19	4.3
Grade	Freshman	42	9.6
	Sophomore	155	35.4
	Junior	162	37.0
	Senior	79	18.0

## 4 Discussion

Due to the cross-sectional design of this study, the findings reveal correlational relationships and moderation patterns among variables rather than definitive causal relationships. Theoretical discussions regarding potential causal directions presented below require further confirmation through longitudinal or experimental research.

Grounded in the Compensatory Internet Use Theory and the I-PACE model, this study established a moderated mediation model to elucidate the psychological mechanisms linking social exclusion to mobile phone dependence among college students. The findings not only validate the core tenets of these theories but also extend them by revealing a nuanced role of social self-efficacy. Specifically, our results demonstrate that while self-control acts as a critical mediator, social self-efficacy—typically considered a positive personal resource—paradoxically strengthens the adverse effects of social exclusion. This underscores the importance of considering contextual factors when applying the I-PACE model, suggesting that certain “Person” factors can operate as vulnerability amplifiers rather than protective buffers under specific environmental stressors like social exclusion.

### 4.1 Direct effect of social exclusion on mobile phone dependence

The results show that social exclusion is significantly and positively associated with mobile phone dependence. In other words, college students who are more socially isolated are more prone to become dependent on their phones. This finding is consistent with H1 and aligns with prior research showing that social exclusion is significantly and positively associated with mobile phone dependence (Xu et al., 2021). The “compensation theory of internet use” is highly supported by this conclusion. Individuals experiencing social exclusion and whose relational needs and sense of belonging are not met in in-person social interactions are more likely to use mobile phones and online socializing to make up for these psychological shortcomings (Hu et al., 2017). Smartphones are essential tools for people to deal with the psychological distress brought on by social exclusion and look for alternative forms of fulfillment because of their anonymity, convenience, and wealth of social resources. This finding aligns with prior research. According to Leary and Baumeister ‘s (1995) need for belonging theory, people who are rejected by a group have a strong need to build relationships with other people in order to satiate their need for belonging. Nonetheless, some people who are socially ostracized display avoidance and social disengagement. As a result, those who are socially excluded are more likely to use their phones for online socializing in order to build connections and interpersonal relationships, which increases their risk of becoming dependent on their phones. Beyond its impact on mobile phone dependence, social exclusion has been shown to influence other psychological processes, such as time perception and subsequent behavioral decisions (Jeon and Cho, 2025). Therefore, it is essential to cultivate positive interpersonal relationships and foster a sense of community belonging in students’ daily lives. Greater attention should be paid to students’ physical and mental health, and timely support should be provided to mitigate the detrimental effects of social exclusion.

#### 4.1.1 Alternative explanations and causal direction

Although our findings align with theoretical models positing social exclusion as a predictor of mobile phone dependence through self-control, the cross-sectional design precludes definitive conclusions regarding causal direction. Several alternative explanations warrant consideration.

Reverse causality. It is possible that mobile phone dependence leads to reduced offline social interaction, thereby increasing the likelihood of social exclusion or perceptions of rejection. Individuals who overuse mobile phones may face fewer face-to-face social opportunities, potentially resulting in social skill deterioration and actual or perceived peer rejection. Longitudinal studies employing cross-lagged panel designs are needed to disentangle these bidirectional possibilities (e.g., Marino et al., 2018).

Third-party variable explanation. Unmeasured variables may account for the observed association. For instance, depression correlates with both heightened sensitivity to social rejection and problematic mobile phone use (Elhai et al., 2021). Social anxiety may similarly predispose individuals to perceive rejection and rely on mobile phones for socially safer online interactions (Marino et al., 2018). Rejection sensitivity—the tendency to anxiously anticipate and readily perceive rejection—may also plausibly explain both higher reported exclusion and greater phone dependence (Downey and Feldman, 1996). Future research should incorporate these potential confounders as covariates or test them as alternative mediators to determine whether proposed models hold beyond these related constructs.

### 4.2 The mediating role of self-control

After establishing the association between social exclusion and mobile phone dependence, our analysis further revealed that self-control partially mediates this relationship. Specifically, social exclusion is negatively associated with self-control, which in turn is positively associated with mobile phone dependence. This finding aligns with prior research identifying self-control as a key factor in problematic mobile phone use (Yao, 2023).

From a theoretical perspective, this mediating effect can be understood through the I-PACE model (Brand et al., 2019) and the finite resource model of self-control (Baumeister et al., 1998; Baumeister et al., 2005). These frameworks suggest that social exclusion may undermine self-control by depleting cognitive and emotional regulatory resources. However, given this study’s cross-sectional design measuring trait self-control rather than momentary resource depletion, this interpretation remains speculative. Future research should employ experimental paradigms directly manipulating resource depletion or utilize state self-control measures to test this mechanism. Consistent with this explanation, experimental research indicates that social rejection impairs self-regulation (Baumeister et al., 2005). However, it is important to note that the cross-sectional design of this study did not directly measure cognitive resource depletion, emotional arousal, or self-exhaustion. Therefore, while our findings align with this resource depletion interpretation, they do not provide direct evidence for this mechanism. Alternative explanations cannot be ruled out; for instance, unmeasured variables such as neuroticism or negative emotionality may predispose individuals to both perceive greater social exclusion and exhibit poorer self-control, leading to increased compensatory mobile phone use.

This model suggests that interventions aimed at enhancing self-control, such as mindfulness or effortful control training, may help reduce the risk of mobile phone dependence among socially excluded individuals. Nevertheless, future research should employ experimental or longitudinal designs to directly measure cognitive load and emotional arousal in order to test the causal pathways proposed here.

Longitudinal studies in recent years have further confirmed the pivotal role of self-control in problematic mobile phone use, indicating that lower trait self-control predicts increased dependence over time (e.g., Du et al., 2021; Busch and McCarthy, 2021). These findings underscore the importance of targeting self-control in preventive interventions.

### 4.3 The moderating role of social self-efficacy: amplifying vulnerability

Our findings indicate that social self-efficacy moderates both the relationship between social exclusion and self-control, as well as the direct relationship between social exclusion and mobile phone dependence, consistently supporting the amplification hypothesis over the buffering hypothesis. This model contributes to the ongoing theoretical debate regarding the functioning of personal resources under interpersonal stress and aligns with recent international research emphasizing the complexity of individual responses to social exclusion (e.g., Zhang X. et al., 2025, Zhang J. et al., 2025; Dembling et al., 2025; Büttner et al., 2025).

#### 4.3.1 Moderation of the relationship between social exclusion and self-control

**Figure 4:**
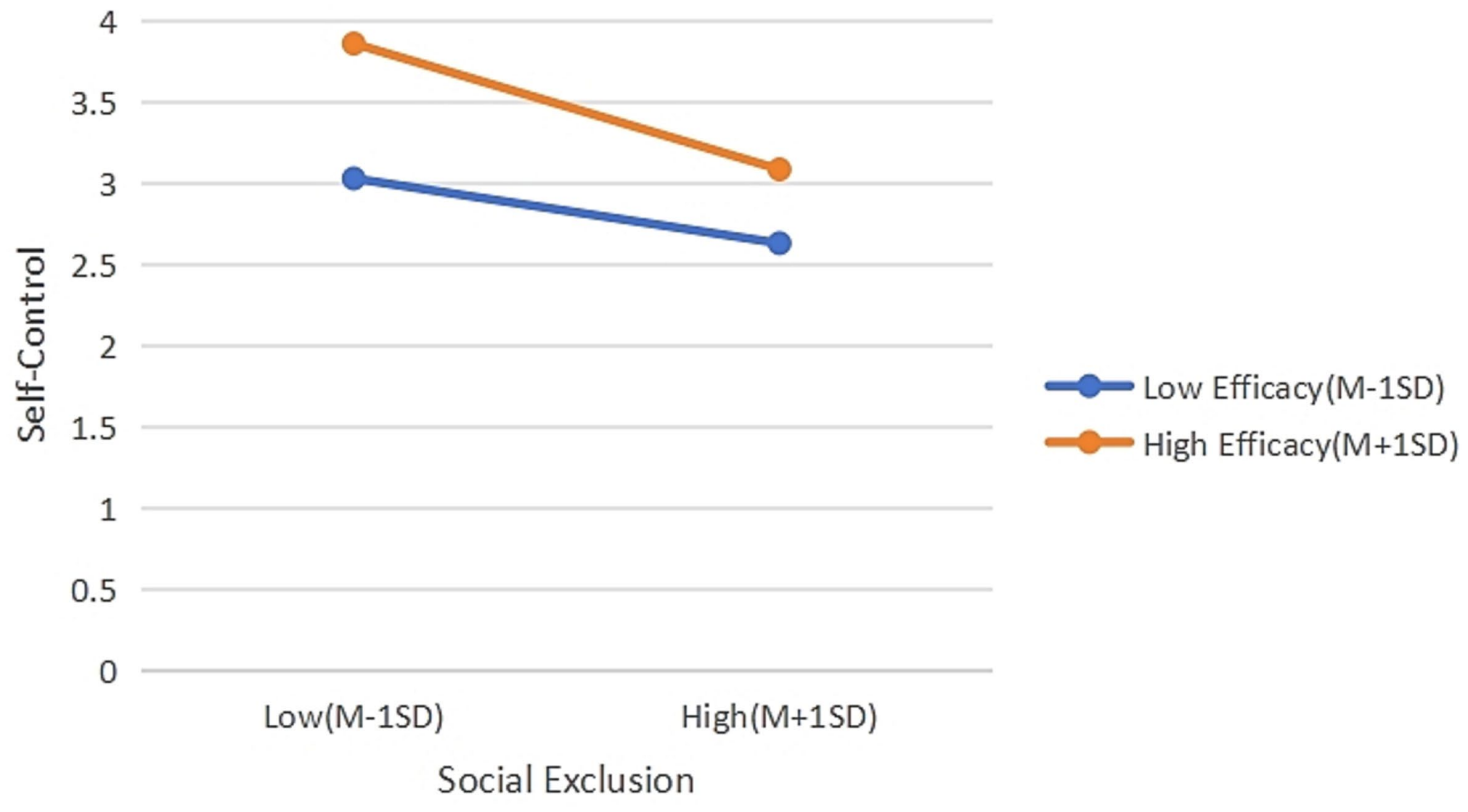
Moderating effect of social self-efficacy between social exclusion and self-control.

As shown in Table 12 and Figure 4, social self-efficacy significantly moderated the association between social exclusion and self-control, with the negative association being stronger among individuals with high SSE. This finding challenges the widely held assumption in Social Cognitive Theory (Bandura, 1997) that self-efficacy universally protects individuals from adversity. However, the notion that personal resources can sometimes amplify rather than buffer stress aligns with recent research on individual differences in responses to social exclusion. For instance, Büttner et al. (2025) demonstrated that narcissists—individuals with heightened expectations of social admiration—exhibit stronger negative reactions to rejection. Similarly, Wu et al. (2025) found that self-concept moderates the impact of rejection on malicious creativity, highlighting the role of how individuals perceive their relationships with others. Neuroimaging evidence further supports this perspective: Zhang X. et al. (2025), Zhang J. et al. (2025) showed that neural responses to social rejection vary by personality traits, while Dembling et al. (2025) found physiological indicators like resting blood pressure correlate with neural sensitivity to exclusion. Collectively, these studies underscore the importance of considering individual-level characteristics when understanding social exclusion reactions.

In the context of the present study, we propose that the amplifying effect of social self-efficacy can be understood through the lens of Expectancy Violation Theory. Individuals with high social self-efficacy hold stronger expectations of social acceptance. When these expectations are violated by social rejection, they may experience heightened disappointment and cognitive dissonance (Festinger, 1957), which can deplete self-regulatory resources and impair self-control (Baumeister et al., 2005). This interpretation aligns with rejection sensitivity research—which shows that individuals with a strong desire for social acceptance are more vulnerable to perceived rejection (Downey and Feldman, 1996; Gao et al., 2017)—and resonates with the broader “too much of a good thing” effect (Pierce and Aguinis, 2013). Thus, our findings do not contradict existing theory but rather extend it by identifying a specific boundary condition—social rejection—under which a typically protective resource (social self-efficacy) may paradoxically induce vulnerability. This interpretation aligns with recent neuroimaging evidence indicating that individuals possessing certain personality traits exhibit heightened neural responses to social rejection (Zhang X. et al., 2025, Zhang J. et al., 2025; Büttner et al., 2025). Furthermore, research demonstrates that neural and physiological responses to rejection are moderated by individual characteristics (Dembling et al., 2025), suggesting that personal traits can shape reactions to interpersonal stressors.

One possible theoretical explanation draws on the Expectancy Violation Theory: Individuals with high social self-efficacy may hold higher expectations for social acceptance; when these expectations are violated, they may experience greater disappointment, thereby increasing cognitive and emotional burdens and impairing self-regulation abilities (Baumeister et al., 2005). However, we emphasize that this interpretation is speculative, as we did not directly measure cognitive load, emotional arousal, or expectation violation. Other mechanisms, such as rumination or heightened sensitivity to social feedback, cannot be ruled out. Future research should incorporate direct measures of cognitive load, physiological indicators (e.g., heart rate variability, cortisol), or experimental manipulations to rigorously test these pathways.

#### 4.3.2 Moderation of the direct relationship between social exclusion and mobile phone dependence

Social self-efficacy also moderated the direct association between social exclusion and mobile phone dependence, with the positive correlation being stronger for individuals with high SSE. This finding further challenges the buffering perspective and supports the amplification hypothesis. Beyond the resource-depletion mechanism that operates through self-control, high-SSE individuals, driven by the expectation of rapid relief from expectation-violation distress, may turn to online platforms where they can leverage their social skills to obtain instant positive feedback (e.g., likes, comments). This interpretation aligns with FoMO research (Elhai et al., 2020) and social comparison studies (Valkenburg et al., 2021), consistent with the notion that individuals with strong social skills may be more sensitive to online social rewards. Research on the bystander effect and social evaluation indicates that even indirect social feedback can influence emotional responses (Poon and Chan, 2025), highlighting the pervasiveness of social evaluation in digital environments.

Recent empirical studies further indicate that individuals with stronger social skills are more sensitive to online social feedback, which may reinforce compensatory use following social exclusion (Faelens et al., 2021; Valkenburg et al., 2022). These findings underscore the necessity for interventions to simultaneously address both online and offline social dynamics.

#### 4.3.3 The dual role of social self-efficacy

Importantly, our findings reveal a dual role of social self-efficacy, which can be understood through a resource-situation matching framework. In the absence of acute social threat, SSE functions as a protective personal resource: individuals with high SSE report greater self-control and lower mobile phone dependence, consistent with Social Cognitive Theory (Bandura, 1997; de Ridr et al., 2012). However, when confronted with social exclusion—a situation directly challenging their positive self-perception—individuals with high SSE may experience heightened conflict between their self-perceived social competence and the reality of rejection. This conflict, predicted by Expectancy Violation Theory, may trigger cognitive dissonance and emotional distress, thereby depleting self-regulatory resources and increasing vulnerability to maladaptive coping (e.g., compulsive mobile phone use). Thus, SSE serves as an adaptive resource in most contexts but may become a risk factor when core expectations are violated. This interpretation aligns with the broader principle of the “too much of a good thing” effect (Pierce and Aguinis, 2013) and underscores the importance of considering person-situation interactions in understanding digital addiction. This dual-function conceptualization resonates with research on the situational dependence of personal resources (Pierce and Aguinis, 2013) and the multifaceted nature of self-regulation (de Ridr et al., 2012). It suggests that the role of personal characteristics in digital addiction critically depends on the match or mismatch between resources and stressful situations.

#### 4.3.4 Practical implications

These findings hold specific implications for intervention design. Given the protective main effect of SSE, broadly enhancing social self-efficacy through social skills training remains valuable. However, interventions should specifically target individuals with high SSE who have recently experienced or are at risk of social exclusion. For these individuals, enhancing general self-efficacy alone may be insufficient; they may require coping strategies for handling rejection that violates expectations. For individuals with low SSE, interventions may focus on building social confidence and providing structured opportunities for positive social engagement. It is important to emphasize that these recommendations are particularly relevant for individuals with high SSE in contexts of social exclusion; in the absence of exclusion, high SSE is typically adaptive and should be supported.

Although the interaction effects reached statistical significance, their effect sizes were modest (Δ*R*^2^ = 1.5% for the social exclusion → self-control path; standardized coefficient for the social exclusion → mobile phone dependence path = 0.09). This suggests that while the moderating role of social self-efficacy is theoretically intriguing, its practical significance may be limited. Replication studies with larger, more diverse samples are needed to confirm the robustness of these findings and assess their generalizability. Therefore, practical implications derived from these interaction effects should be treated with caution.

### 4.4 Theoretical implications

This study makes two specific contributions to understanding digital dependence within the I-PACE framework (Brand et al., 2019).

First, we identified boundary conditions for the protective role of personal resources. The I-PACE model traditionally conceptualizes personal factors (P) as stable traits predisposing individuals to addictive behaviors, but it inadequately explains how these factors interact with situational stressors. Our findings indicate that the effect of social exclusion on mobile phone dependence, mediated through self-control (conceptualized as an executive function resource within the E dimension), depends on levels of social self-efficacy (a P factor). This identifies a theoretically important boundary condition: personal resources that typically exert a protective effect may fail to buffer (or may even amplify) risk under specific stress conditions. This extends the I-PACE model by specifying person-situation interactions that moderate pathways between executive function and addictive behaviors. It is crucial to emphasize, however, that actual psychological processes may be more complex than mediation models suggest. Self-control may engage in bidirectional or interactive effects with other affective and cognitive processes (A, C), and our operationalization as a stable disposition merely offers an empirical perspective for understanding these dynamic processes.

Second, we refined the conceptualization of personal resources within addiction models. Unlike views treating personal resources as uniformly protective or uniformly risk-amplifying, our findings propose a more nuanced “dual-function” model: social self-efficacy simultaneously exhibits protective main effects and situational amplification effects. This dual-function conceptualization challenges assumptions of resource uniformity and aligns with recent international research on the complexity of social exclusion effects. For example, research indicates that neural and physiological responses to rejection vary across individuals based on traits such as personality characteristics (Büttner et al., 2025; Zhang X. et al., 2025, Zhang J. et al., 2025), and these responses are jointly moderated by contextual and dispositional factors (Dembling et al., 2025). Collectively, these studies underscore the importance of accounting for individual differences when understanding the consequences of social exclusion. Our findings extend this research trajectory by demonstrating that even seemingly positive resources like social self-efficacy can produce contradictory effects depending on the context.

### 4.5 Practical implications

Our findings hold specific implications for intervention design. First, given the protective main effect of SSE, broadly enhancing social self-efficacy through social skills training and positive social experiences remains a valuable preventive goal. However, our moderation findings suggest that interventions should specifically target individuals with high SSE who have recently experienced or are at risk of social exclusion. For these individuals, enhancing general self-efficacy alone may be insufficient and could even increase vulnerability if not accompanied by strategies to address expectations of social rejection.

Specifically, interventions for high-SSE individuals facing exclusion should: (a) normalize rejection experiences, even for socially adept individuals, to reduce self-concept threat; (b) provide cognitive restructuring techniques to address discrepancies between self-perceived competence and rejection experiences; (c) teach adaptive coping strategies to redirect social confidence toward rebuilding offline relationships rather than retreating into online compensation; (d) monitor mobile phone usage patterns following exclusion events to prevent the development of compensatory dependence cycles.

For low SSE individuals, interventions may focus on building social confidence and providing structured positive social engagement opportunities, as these individuals may be more vulnerable to rejection effects but exhibit lower reactivity to the specific mechanisms we identified.

It is important to emphasize that our intervention recommendations are particularly applicable to high SSE individuals in contexts of social exclusion; in the absence of exclusion, high SSE is typically adaptive and should be supported.

Notably, our exploratory analysis revealed significant differences between rural and urban students in social exclusion, self-control, social self-efficacy, and mobile phone dependence. Although we statistically controlled for these demographic variables in the main model, the observed disparities suggest that the associative mechanisms linking social exclusion to mobile phone dependence may vary across different groups. For instance, rural students may encounter distinct social stressors or have limited access to offline social resources, which could either amplify or buffer the effects of exclusion. Future research should test whether this moderated mediation model holds across urban and rural subsamples and explore contextual factors (e.g., social integration, digital infrastructure) that may explain these differences. Such insights could inform tailored interventions based on student backgrounds.

### 4.6 Limitations and future directions

Several limitations warrant consideration and provide directions for future research.

First, cross-sectional designs preclude causal inference. Although our theoretical model specifies social exclusion as a predictor and mobile phone dependence as an outcome, a bidirectional relationship is plausible. Mobile phone dependence may reduce offline social engagement, potentially increasing actual or perceived exclusion. Experimental paradigms manipulating social exclusion (e.g., Cyberball) and tracking subsequent phone usage could establish causal effects. Multi-wave longitudinal designs could test reciprocal relationships and identify temporal precedence.

Second, self-report measures introduce potential biases. While our CFA analysis indicates common method variance is not a severe issue, self-reports of social exclusion, self-control, and phone use may be influenced by recall bias, social desirability, or current emotional states. Future research should incorporate objective measures (e.g., actual phone usage data from device logs, behavioral measures of self-control) and multi-source reporting (e.g., peer nominations of exclusion) to triangulate findings.

Third, while we theoretically proposed cognitive resource depletion and cognitive dissonance as underlying mechanisms for the observed effects, we did not directly measure these processes. Future research should incorporate direct measures of cognitive load, self-exhaustion, stress physiological indicators (e.g., heart rate variability, cortisol), or cognitive dissonance measures (e.g., discomfort ratings, inconsistency detection tasks) to rigorously test these causal pathways.

Fourth, the small effect sizes of the interaction terms (*β* = −0.14 and 0.09) warrant caution in interpretation. While statistically significant and theoretically meaningful, these effects fall within the small-to-medium range. This aligns with meta-analytic findings that interaction effects in social science research are typically modest (Aguinis et al., 2021). Robust conclusions can only be drawn after replication studies with larger, more diverse samples.

Fifth, we did not measure specific online activities or the social feedback participants received. While we theoretically proposed that individuals with high SSE may be more susceptible to the reinforcing effects of online social feedback (e.g., likes, comments), we did not directly test this mechanism. Future research should employ experiential sampling to capture real-time dynamics of online behavior following social exclusion, examining whether high-SSE individuals engage in more proactive social media use, receive greater positive feedback, and whether such feedback reinforces subsequent mobile phone usage.

Sixth, the sample was limited to Chinese university students, which may restrict the generalizability of the findings. Cultural factors—such as collectivism, face concerns, and mobile phone usage norms—may influence the observed relationships. Cross-cultural studies are needed to examine whether this moderated mediation model holds in individualistic cultures or across different age groups.

Seventh, we observed significant differences between urban and rural students on key variables. Although we statistically controlled for these demographic factors, the mechanisms underlying these differences remain unclear. Future research should examine whether the model holds equally across urban and rural subgroups and identify contextual factors (e.g., social integration, digital infrastructure) that may explain these disparities.

Eighth, we did not include potentially important third variables such as depression, social anxiety, or rejection sensitivity, which have been shown to correlate with both social exclusion and mobile phone dependence. Future research should incorporate these constructs as covariates or alternative mediators to test the robustness of the proposed model.

### 4.7 Conclusion

This study examined the associations among social exclusion, self-control, social self-efficacy, and mobile phone dependence in a sample of Chinese college students. Key findings are as follows:

(1) Social exclusion was positively associated with mobile phone dependence;
(2) Self-control partially mediated this association;
(3) Social self-efficacy moderated the relationship between social exclusion and self-control: the negative association was stronger among students with high social self-efficacy;
(4) Social self-efficacy also moderated the direct association between social exclusion and mobile phone dependence: the positive association was stronger among students with high social self-efficacy.

These findings underscore the importance of considering mediating and moderating mechanisms when understanding mobile phone dependence and suggest that interventions may need to be tailored based on individuals’ levels of social self-efficacy. However, due to the cross-sectional design, causal conclusions cannot be drawn, and repeated validation using longitudinal data is required.

## Data Availability

The raw data supporting the conclusions of this article will be made available by the authors, without undue reservation.
