# Supplementary material for: Social exclusion and mobile phone dependence in college students: a moderated mediation model of self-control and social self-efficacy
Source: Front Psychol. 2026 Apr 13;17:1752086. doi: 10.3389/fpsyg.2026.1752086 (PMC13111211; doi:10.3389/fpsyg.2026.1752086)
Supplement: Supplementary file 1 [file Table_1.DOCX]

**Appendices**

**The questionnaire contents are all in Chinese and the Chinese version is provided below the English version.**

Dear Student,

Hello! This is an anonymous survey questionnaire designed to study the impact of social exclusion on college students' mobile phone dependency. There are no right or wrong answers, and your responses are crucial to the success of this research. Please complete the questionnaire honestly based on your actual circumstances. All responses will be kept strictly confidential. Thank you for your support and cooperation!

1.Gender: ① Male ② Female

2. Year of Study: ① Freshman ② Sophomore ③ Junior ④ Senior

3. Place of Origin: ① Rural ③ Urban

**Questionnaire I: College Student Social Exclusion Questionnaire**

Instructions: Social exclusion is common—for instance, when classmates plan outings without inviting you; others refuse to lend you course notes; or people treat you coldly. These are all forms of exclusion. Please recall specific instances in your past where you experienced exclusion (e.g., rejection, isolation, or being ignored by family, friends, classmates, teachers, strangers, or groups). Close your eyes and revisit those situations, making them feel as vivid as if they were happening right now. Do not worry about others' opinions; answer based solely on your own recollections.

| Item | Never | Rarely | Sometimes | Often | Always |
| --- | --- | --- | --- | --- | --- |
| 1.When others teased or played around, they intentionally or unintentionally excluded me | 1 | 2 | 3 | 4 | 5 |
| 2. I became the target of malicious pranks | 1 | 2 | 3 | 4 | 5 |
| 3.Others wouldn't share their feelings or experiences with me | 1 | 2 | 3 | 4 | 5 |
| 4.People spoke ill of me behind my back, influencing others' opinions of me | 1 | 2 | 3 | 4 | 5 |
| 5.My mistakes are met with jeering or harsh criticism | 1 | 2 | 3 | 4 | 5 |
| 6.People often point out my errors or oversights and report them to others | 1 | 2 | 3 | 4 | 5 |
| 7.When I join a group conversation, it goes cold | 1 | 2 | 3 | 4 | 5 |
| 8.When I might embarrass myself or make a mistake, others just wait to see me fail | 1 | 2 | 3 | 4 | 5 |
| 9.Others mock my weaknesses, hurting my feelings | 1 | 2 | 3 | 4 | 5 |
| 10. I receive unkind looks for no apparent reason | 1 | 2 | 3 | 4 | 5 |
| 11.When I'm feeling down, I don't get any comfort or reassurance from others. | 1 | 2 | 3 | 4 | 5 |
| 12.Even when I try hard to improve relationships, I don't get positive responses. | 1 | 2 | 3 | 4 | 5 |
| 13.Even if we know each other, people don't greet me proactively. | 1 | 2 | 3 | 4 | 5 |
| 14.People respond to my questions or requests with impatience and dismissiveness | 1 | 2 | 3 | 4 | 5 |
| 15.My attempts to strike up conversations rarely get enthusiastic responses | 1 | 2 | 3 | 4 | 5 |
| 16.Others rarely notice me and know little about my circumstances | 1 | 2 | 3 | 4 | 5 |
| 17.Others intentionally or unintentionally create physical distance from me | 1 | 2 | 3 | 4 | 5 |
| 18.During conversations, no matter what topic I bring up, others rarely engage | 1 | 2 | 3 | 4 | 5 |
| 19.My interactions with others seem difficult to deepen or prolong | 1 | 2 | 3 | 4 | 5 |

**Questionnaire 2: Chinese Version Mobile Phone Dependency Index Scale**

Instructions: The following statements pertain to your mobile phone usage. There are 17 items in total. Each item corresponds to one answer. Please respond based on your actual experiences over the past year.

| Item | Never | Rarely | Sometimes | Often | Always |
| --- | --- | --- | --- | --- | --- |
| 1.You have been told you spend too much time on your phone | 1 | 2 | 3 | 4 | 5 |
| 2.Your friends and family complain about your constant phone use | 1 | 2 | 3 | 4 | 5 |
| 3.You have tried to hide how much time you spend on your phone from others | 1 | 2 | 3 | 4 | 5 |
| 4.You find you spend more time on your phone than you intended | 1 | 2 | 3 | 4 | 5 |
| 5.You always feel like you don't have enough time to use your phone | 1 | 2 | 3 | 4 | 5 |
| 6.You have tried to spend less time on your phone but failed | 1 | 2 | 3 | 4 | 5 |
| 7.You sacrifice sleep time to use your phone | 1 | 2 | 3 | 4 | 5 |
| 8.When away from your phone beyond a certain distance, you feel anxious about missing calls or messages from friends | 1 | 2 | 3 | 4 | 5 |
| 9.You feel anxious if you go without checking messages, browsing the internet, or turning on your phone for a period of time | 1 | 2 | 3 | 4 | 5 |
| 10. You find it difficult to turn off your phone | 1 | 2 | 3 | 4 | 5 |
| 11.You feel lost without your phone | 1 | 2 | 3 | 4 | 5 |
| 12.You use your phone to communicate with others when feeling isolated | 1 | 2 | 3 | 4 | 5 |
| 13.You have used your phone to communicate with others when feeling lonely | 1 | 2 | 3 | 4 | 5 |
| 14.When feeling down, have you used your phone to alleviate those feelings? | 1 | 2 | 3 | 4 | 5 |
| 15.Have you neglected other responsibilities due to phone use, causing problems? | 1 | 2 | 3 | 4 | 5 |
| 16.Is time spent on your phone a direct cause of reduced productivity? | 1 | 2 | 3 | 4 | 5 |
| 17.Sometimes you prefer using your phone over addressing more urgent matters | 1 | 2 | 3 | 4 | 5 |

**Questionnaire 3: College Student Self-Control Scale**

Instructions: The following statements describe feelings in your life. Please select based on your personal experience.

| Item | Strongly Disagree | Disagree | Undecided | Agree | Strongly Agree |
| --- | --- | --- | --- | --- | --- |
| 1.I can effectively resist temptations. | 1 | 2 | 3 | 4 | 5 |
| 2.It's hard for me to break bad habits. | 1 | 2 | 3 | 4 | 5 |
| 3.I am lazy. | 1 | 2 | 3 | 4 | 5 |
| 4.I do things that bring me pleasure but are harmful to myself. | 1 | 2 | 3 | 4 | 5 |
| 5.People believe I can stick to action plans. | 1 | 2 | 3 | 4 | 5 |
| 6.Getting up in the morning is difficult for me. | 1 | 2 | 3 | 4 | 5 |
| 7.People say I'm impulsive. | 1 | 2 | 3 | 4 | 5 |
| 8.I spend money too freely. | 1 | 2 | 3 | 4 | 5 |
| 9.I get so emotionally excited that I can't control myself. | 1 | 2 | 3 | 4 | 5 |
| 10.Many things I do are because of a momentary impulse. | 1 | 2 | 3 | 4 | 5 |
| 11.People say I have ironclad self-control | 1 | 2 | 3 | 4 | 5 |
| 12.Sometimes I get distracted by fun things and can't finish tasks on time. | 1 | 2 | 3 | 4 | 5 |
| 13. I have trouble concentrating. | 1 | 2 | 3 | 4 | 5 |
| 14.I can work efficiently toward a long-term goal. | 1 | 2 | 3 | 4 | 5 |
| 15.Sometimes I can't resist doing things even when I know it's wrong. | 1 | 2 | 3 | 4 | 5 |
| 16.I often act without thinking things through. | 1 | 2 | 3 | 4 | 5 |
| 17.I get angry too easily. | 1 | 2 | 3 | 4 | 5 |
| 18.I frequently disturb others. | 1 | 2 | 3 | 4 | 5 |
| 19. I sometimes drink (or surf the internet) excessively. | 1 | 2 | 3 | 4 | 5 |

**Questionnaire 4: Social Self-Efficacy Scale**

Instructions: Please answer based on your first impression after reading each statement without overthinking.

| Item | Not at all confident | Somewhat not confident | Unsure | Somewhat confident | Completely confident |
| --- | --- | --- | --- | --- | --- |
| 1. Initiate conversation with someone I don't know well. | 1 | 2 | 3 | 4 | 5 |
| 2.Expressing your views on a topic of interest to a group discussing it. | 1 | 2 | 3 | 4 | 5 |
| 3.Collaborating on a project or activity with unfamiliar people in a school club. | 1 | 2 | 3 | 4 | 5 |
| 4.Making someone you've just met feel comfortable joining your circle of friends. | 1 | 2 | 3 | 4 | 5 |
| 5. Finding friends to spend your weekends with. | 1 | 2 | 3 | 4 | 5 |
| 6.Putting yourself in new, unfamiliar social settings. | 1 | 2 | 3 | 4 | 5 |
| 7.Volunteering to help someone lead a group or organization. | 1 | 2 | 3 | 4 | 5 |
| 8.Volunteering to help someone organize an event. | 1 | 2 | 3 | 4 | 5 |
| 9.Share a past enjoyable experience with a group of people. | 1 | 2 | 3 | 4 | 5 |
| 10.Find friends to have lunch with. | 1 | 2 | 3 | 4 | 5 |
| 11. Join a table where people are already seated and conversing. | 1 | 2 | 3 | 4 | 5 |
| 12.Attend a gathering or social event where you may not know anyone. | 1 | 2 | 3 | 4 | 5 |
| 13.Ask others for help when you need it. | 1 | 2 | 3 | 4 | 5 |
| 14.Make friends with people your own age. | 1 | 2 | 3 | 4 | 5 |
| 15.Ask someone you're interested in out on a date. | 1 | 2 | 3 | 4 | 5 |
| 16.Making friends within a circle of people who already know each other. | 1 | 2 | 3 | 4 | 5 |
| 17.Inviting someone out again, even if your first invitation was unsuccessful | 1 | 2 | 3 | 4 | 5 |
| 18.Taking the initiative to call someone you've met and want to get to know better. | 1 | 2 | 3 | 4 | 5 |
